# The Abortive Treatment of Coryza

**Published:** 1892-08-13

**Authors:** 


### The Practitioner's Mirror and Retrospect.

[*The Editor will be glad to receive offers of co-operation and contributions from members of the profession. All letters should be addressed to* The Editor, The Lodge, Porchester Square, London, W.]

### MEDICINE.

### THE ABORTIVE TREATMENT OF CORYZA.

There are many facts which leave little doubt that acute nasal catarrhs or common colds in the head are directly dependent on micro-organisms. A vulnerable mucous membrane is perhaps rendered susceptible by more or less sudden lowering of the surface temperature, and the chill therefore leads to the successful inoculation. Individuals, even in the same family, vary greatly in their susceptibility; but one having caught the cold, it would appear that the virulence of the micro-organism is increased by its growth in its suitable habitat, with the result that the less susceptible are all in turn inoculated, and, once started by one member of the family, the affection "is sure to go all round." There is about as much clinical evidence to support this view that these acute nasal catarrhs are analagous to the exanthemata in their nature as that pneumonia is a zymotic disease. There is an incubation period of about one or two days, with a general feeling of malaise, and a peculiar dryness in the soft palate, followed by the acute symptoms of nasal and frontal-sinus catarrh, with or without some feverishness.

At any rate, practical experience has shown that it is possible to abort a cold if it be taken in time, and most of the remedies that have proved successful are local stimulants to the nasal and pharyngeal mucosa or local germicides. Thus Lederman, of New York,* has found the following mode of treatment very beneficial during the congestive stage of an acute nasal catarrh. The nasal chambers are sprayed with any of the antiseptic solutions, Seiler's preferred, until they are sufficiently cleansed, and then the following solution is used:

℞ Cocaine,
Menthol, aa grs xx;
Benzoinol, f℥ii M.
ft. solutio.

The feeling of fulness in the nose, associated with the dull frontal headache, is no doubt the result of extreme congestion. For the relief of these symptoms, cocaine has been found useful, though its effect soon passes off, and the patient is left in the same uncomfortable position as before. To obviate this, menthol is added to the solution. Menthol is a local anæsthetic, and, in combination with cocaine, it keeps up the local depletion, and leaves a pleasant coolness in the nasal chambers, enabling the patient to breathe through the natural passages. Benzionol is used in this solution as a menstruum, on account of its bland and unirritating qualities, and freedom from unpleasant odour.

Another remedy which, according to a recent number of the *Pharmaceutishe Post*, has been employed with much success in treating nasal catarrh, is the inhalation of fumes of alpha-oxynaphthæic acid crystals. This is formed by the action of carbonic acid under high temperature, and pressure on alpha-sodium naphthol, and it is claimed to possess five times the antiseptic power of salicylic acid. About fifteen grains of the white crystalline powder is gently warmed, and after inhaling, the catarrh at first becomes markedly increased from the irritating effects of the remedy, but after a few hours it undergoes rapid improvement. Ordinarily, it is claimed that one employment of this remedy is sufficient.

Capitant† recommends the insufflation of the following powder into each nostril, for the aborting of an acute coryza in the early stages:—

℞ Salol grs. xv.
Acid salicylic, grs. iii.
Acid tannic, grs. ii.
Acid boric (pulv.) ʒi.

This treatment should not be continued for more than half a day, as the carbolic acid set free from the salol will injure the nose. After this powder, one composed of powdered talc and boric acid may be employed, or the following may be used in place of any of those given, and is equally satisfactory and more safe:—

℞ Pulv. talc, gr. lxxv.
Antipyrin, gr. xv.
Acid boric (pulv.), gr. xxx.
Acid salicylic, gr. iv.

A pinch of this may frequently be used without any of the disagreeable symptoms caused by the first prescription given.

Great relief will be obtained, and not infrequently a catarrh may be aborted by a few pinches of Beehag's menthol snuff:—

Menthol, 1 part.
Acid boracic, 2 parts.
Ammon chloride, 3 parts.

Of course it is unnecessary to refer to the many old-established and more or less efficient household remedies, which may be useful adjuncts in the treatment when bed-time arrives, but the use of some convenient powders, such as we have referred to, will often do all that is necessary without involving any disagreeable or impossible conditions.

* *Brooklyn Medical Journal*, December, 1891.

† *La Méd.*, Mod. Nov. 12th, 1891; *Theraputic Gazette*, Feb. 15th, 1892.

